# Plant-derived differences in the composition of aphid honeydew and their effects on colonies of aphid-tending ants

**DOI:** 10.1002/ece3.1277

**Published:** 2014-10-03

**Authors:** Elizabeth G Pringle, Alexandria Novo, Ian Ableson, Raymond V Barbehenn, Rachel L Vannette

**Affiliations:** 1 Michigan Society of Fellows, University of MichiganAnn Arbor, Michigan, 48109; 2 Department of Ecology and Evolutionary Biology, University of MichiganAnn Arbor, Michigan, 48109; 3 School of Natural Resources and Environment, University of MichiganAnn Arbor, Michigan, 48109; 4 Department of Molecular, Cellular and Developmental Biology, University of MichiganAnn Arbor, Michigan, 48109; 5 Department of Biology, Stanford UniversityStanford, California, 94305

**Keywords:** *Aphis nerii*, *Asclepias* spp., carbohydrate, cardenolides, *Linepithema humile*, milkweed, phloem chemistry, tritrophic interactions

## Abstract

In plant–ant–hemipteran interactions, ants visit plants to consume the honeydew produced by phloem-feeding hemipterans. If genetically based differences in plant phloem chemistry change the chemical composition of hemipteran honeydew, then the plant's genetic constitution could have indirect effects on ants via the hemipterans. If such effects change ant behavior, they could feed back to affect the plant itself. We compared the chemical composition of honeydews produced by *Aphis nerii* aphid clones on two milkweed congeners, *Asclepias curassavica* and *Asclepias incarnata*, and we measured the responses of experimental *Linepithema humile* ant colonies to these honeydews. The compositions of secondary metabolites, sugars, and amino acids differed significantly in the honeydews from the two plant species. Ant colonies feeding on honeydew derived from *A. incarnata* recruited in higher numbers to artificial diet, maintained higher queen and worker dry weight, and sustained marginally more workers than ants feeding on honeydew derived from *A. curassavica*. Ants feeding on honeydew from *A. incarnata* were also more exploratory in behavioral assays than ants feeding from *A. curassavica*. Despite performing better when feeding on the *A. incarnata* honeydew, ant workers marginally preferred honeydew from *A. curassavica* to honeydew from *A. incarnata* when given a choice. Our results demonstrate that plant congeners can exert strong indirect effects on ant colonies by means of plant-species-specific differences in aphid honeydew chemistry. Moreover, these effects changed ant behavior and thus could feed back to affect plant performance in the field.

## Introduction

Feedbacks between the community ecology and evolution of organisms are important but remain poorly understood (Strauss et al. 2005; Utsumi 2013). Plant–arthropod interactions are promising systems for studying such feedbacks. Past studies have firmly established that plant species and genotypic diversity affect arthropod communities (Johnson 2008; Cook-Patton et al. 2011) and that herbivores affect plant community membership and fitness (Fine et al. 2004;Johnson et al. 2009). In tritrophic interactions among plants, herbivores, and ants, honeydew-producing hemipteran insects attract ants to plants, and ants reduce the abundances of nonhemipteran herbivores (Styrsky and Eubanks 2007). If genetically determined plant traits influence the quantity or composition of hemipteran honeydew, such traits could affect the fitness and behaviors of honeydew-feeding ant colonies (Cushman 1991). Such effects might go on to structure ecological communities because ants often exert large effects on other arthropods (Hölldobler and Wilson 1990; Wimp and Whitham 2001) and on plants themselves (Moreira et al. 2012).

If plant genetic differences underlie the differential attractiveness of hemipterans, such as aphids, to ants, plant phloem chemistry could represent an important set of traits under diffuse selection. Mooney and Agrawal (2008) demonstrated variation among genotypes of common milkweed plants (*Asclepias syriaca*) in per capita ant attendance of aphids and suggested that such variation could result from genetically determined differences in plant phloem composition. Although aphids modify phloem as it passes through their guts (Douglas 2006), honeydew composition also depends on the host plant (Mittler 1958; Hendrix et al. 1992). In particular, amino acids, sugars, and water-soluble plant defense compounds in hemipteran honeydew reflect those in plant phloem (Molyneux et al. 1990; Douglas 1993; Völkl et al. 1999). In addition, because hemipterans osmoregulate by producing oligosaccharides from simpler phloem sugars (Fisher et al. 1984; Douglas 2006), complex sugar profiles, even if produced by the hemipterans, may also reflect the host plant's chemistry (Fischer et al. 2005).

Ants display distinct nutritional preferences when offered a variety of food sources (Blüthgen and Fiedler 2004) and can regulate nutritional intake at the levels of both the individual forager and the colony (Cassill and Tschinkel 1999; Dussutour and Simpson 2008a). Although predatory arthropods, including ants, have been hypothesized to be nitrogen-limited (Denno and Fagan 2003), recent evidence suggests that ant colony establishment, growth, behavior, and life span depend strongly on carbohydrate availability (Grover et al. 2007; Wilder et al. 2011; Dussutour and Simpson 2012; Shik and Silverman 2013). It has been suggested that oligosaccharides, in particular melezitose, are important for attracting ants to aphid honeydew (Völkl et al. 1999), but many ants prefer sucrose and glucose to nearly all other sugars (Cornelius et al. 1996; Blüthgen and Fiedler 2004), and complex sugars may serve mostly to indicate concentrated sugar resources (Woodring et al. 2004). Ants may also prefer mixtures of sugars and amino acids, such as those that would typically be found in honeydew, to sugar alone (Blüthgen and Fiedler 2004). Although ant preference for low levels of plant secondary compounds in honeydew might be expected (Bristow 1991; Vrieling et al. 1991), this appears never to have been demonstrated experimentally.

Plants and ants could coevolve in interactions mediated by aphids and nonaphid herbivores if (1) the plant's genotype affects aphid honeydew composition; (2) the composition of aphid honeydew affects ant colony fitness; and (3) ants affect plant fitness (either negatively or positively, depending on the relative costs of aphids versus nonaphid herbivores). Evidence for these conditions is incomplete. Field studies have shown that plant genotypic diversity (both within and between species) affects constitutive and induced trait-mediated ant responses to aphids (Mooney and Agrawal 2008; Abdala-Roberts et al. 2012; Moreira et al. 2012), but we do not know what chemical changes underlie these differences in aphid attractiveness. Laboratory studies have demonstrated that access to aphid honeydew positively affects ant colony growth and establishment (Wilder et al. 2011; Shik and Silverman 2013), but we do not know whether ant colonies are affected by the subtle nutritional differences in honeydews derived from different plant genotypes. Finally, hemipteran-tending ants strongly, and often positively, affect plant performance and fitness (Styrsky and Eubanks 2007; Pringle 2014), but nutritional inputs to ant colonies have rarely been explicitly linked to ant behaviors with respect to ant–plant interactions (but see, e.g., Pringle et al. 2011). Such ant behaviors will determine the magnitude and direction of the ants' effects on plants.

In this study, we address these lacunae in a tritrophic laboratory system based on two milkweed plant species, *Asclepias incarnata* L. and *Asclepias curassavica* L. (Apocynaceae). These closely related (Fishbein et al. 2011) congeneric species both host specialized aphids, including *Aphis nerii* Fonscolombe (Helms et al. 2004; Martel and Malcolm 2004). In addition, *A. incarnata* and *A. curassavica* exhibit similarities in both architecture (Martel and Malcolm 2004) and leaf nutrient concentration (Tao et al. 2014) but differ in their concentrations of leaf cardenolides (de Roode et al. 2011). Here we show that there are chemical differences between the honeydews derived from aphid clones on the two plant species and that these differences affect the maintenance, feeding preferences, and behaviors of honeydew-feeding *Linepithema humile* Mayr ant colonies.

## Materials and Methods

### Study system and experimental set-up

We compared plant-derived variation in aphid–ant interactions using two milkweed species, *A. incarnata* and *A. curassavica*, in a controlled, laboratory environment in Ann Arbor, MI. Two experiments were conducted. For both experiments, seeds were purchased from Butterfly Encounters (Dublin, CA). Prior to germination, seeds of both species were washed in 5–10% bleach in water. Seeds of *A. incarnata* were cold stratified at 4°C for 6–7 weeks before germination. Plants were grown in a random block design in a single growth chamber at ∼25°C and a 12L:12D light cycle. Seeds were germinated on wet filter paper for 1 week. Germinated seeds were planted in seedling flats and ultimately in 4-inch pots with autoclaved Metro-Mix 380 potting soil (Sun Gro Horticulture, Seba Beach, Canada). After transfer to pots, plants were fertilized with 50 g/m^2^ 20N:20P:20K MiracleGro® (Marysville, OH) fertilizer in three treatments over 4 weeks (total ≈ 4 g/m^2^ nitrogen).

We compared the chemical compositions of the honeydews produced by *A. nerii* aphids on *A. incarnata* and *A. curassavica* host plants. Aphid populations were clones derived from a single individual collected in Emmett County, MI in September 2011 and reared in the laboratory for >5 generations on the same host plant species to which individuals were transplanted for the experiments. Previous aphid population growth experiments revealed that 2-week total growth was variable between plant species, but that aphid population size per plant aboveground dry mass was consistently higher on *A. incarnata* (data not shown). In addition, aphid populations on both plant species began to crash after ∼2 weeks.

We measured the effects of plant species differences in aphid honeydew on *L. humile* ant colonies. We used *L. humile*, commonly known as the Argentine ant, to measure ant responses because it is an important and well studied invasive species that depends largely on low-nitrogen, plant-derived resources in its introduced U.S. range (Tillberg et al. 2007). *Linepithema humile* has also been reported to tend *A. nerii* aphids under field conditions in California (Bristow 1991). Ant workers and queens were collected at six locations in Rose and San Clemente Canyons, San Diego County, CA (32.8°N, 117.1°W), in September 2013. Throughout this area, *L. humile* forms huge, polygynous colonies that exhibit very low genetic diversity (Tsutsui et al. 2000). Nevertheless, we maintained source colonies separately by collection site, and queens for each experimental colony were matched with workers from the same site. Ants were kept in 38 × 51 × 18 cm polypropylene bins whose sides were lined completely with fluon (Insect-a-Slip, BioQuip, CA). Colonies were provided 10 × 75 mm glass culture tubes for nesting, which were covered in red cellophane, filled halfway with water, and plugged with cotton.

To control ant diet completely and monitor worker behavior more accurately, we prevented ants from nesting in plant soil. We covered plant pots with ∼28 holes/cm chiffon mesh and secured mesh around plant stems using Mortite caulking cord (Thermwell, NJ) and hot glue. Each bin containing an experimental ant colony and aphid-colonized plant(s) was placed beneath a 61-cm table-top light with two T8 lamps on a 12L:12D light cycle. Experiments were conducted in two growth rooms maintained at ∼25°C. In the 6-week forced-diet experiment, bins were switched between rooms once per week, and bins containing *A. incarnata* and *A. curassavica* plants were maintained in alternating order.

To provide the ants with a controlled source of nitrogen, we made a protein-biased artificial diet. The diet was made in an ∼3:1 protein to carbohydrate (p:c) w/w ratio according to a recipe modified from Dussutour and Simpson (2008b). The diet was poured into Petri dishes and stored at 4°C until immediately before it was provided to ant colonies. Source colonies were fed a solution of 20% sucrose in water and the artificial diet ad libitum. In addition to the aphid honeydew, which was mostly carbohydrate, experimental colonies were provided with water in nesting tubes ad libitum (tubes were replaced every 2 weeks) and with 1 g wet mass of the 3:1 p:c artificial diet 3 days per week.

Statistical analyses were conducted in JMP® Pro 10.0 (SAS Institute 2012) and R version 3.0.3 (R Core Team 2014). All values are reported mean ± SE. Models were chosen after checking residuals for normality and homoscedasticity; where appropriate, we used nonparametric tests.

### Honeydew chemical analysis

Honeydew was collected every 2 weeks in the forced-diet experiment (see below) from a total of 36 plants per species, that is, once from each of the three aphid-colonized plants used in each replicate. Honeydew was collected on 0.32 cm^2^ round aluminum disks. Disks were freeze-dried overnight, weighed, and secured on plants beneath aphid congregations with double-sided tape. Honeydew was collected for 48 or 72 h (in the first collection and in the second and third collections, respectively). Disks with honeydew were then freeze-dried overnight, reweighed, and honeydew was washed from disks into 100 μL 9:1 water:methanol. Samples from all collections were pooled to make 6–8 independent replicates per species for cardenolide, sugar, and amino acid analyses (∼0.10, 0.07, and 0.17 mg of honeydew per replicate, respectively). These samples were then evaporated in an Eppendorf Vacufuge® for ∼40–60 min at room temperature and stored at −20°C until analysis.

To measure honeydew cardenolides, samples were resuspended in 150 μL methanol with an internal standard of 0.15 mg/mL digitoxin and analyzed by reverse-phase ultra-high-performance liquid chromatography (UPLC, Waters Inc., Milford, MA). For more details, see Tao et al. (2014). We compared cardenolide polarity, which affects their toxicity to animal consumers (Agrawal et al. 2012), to previously published data on cardenolides in *A. nerii* honeydew (Malcolm 1990) and in the leaves of both host plants (de Roode et al. 2011; Sternberg et al. 2012). We also measured cardenolides in *A. nerii* honeydews from both plant species in a pilot experiment conducted in April 2013; these data are presented for comparison in Appendix 1.

To measure honeydew sugars, samples were resuspended in 50:50 acetonitrile (MeCN):water (H_2_O) and vortexed vigorously. Samples were filtered through a 0.22-μm centrifugal filter (Fisher Scientific). Saccharides were separated by UPLC using a Luna amide column (50 × 2 mm, 3 μm, Phenomenex, Torrance, CA). Each run employed an acetonitrile:water mobile phase beginning with a 4 min isocratic elution at 80:20 MeCN:H_2_O, followed by a 5 min linear gradient ending at 30:70 MeCN:H_2_O, with a 10-min equilibration at initial conditions between samples. Saccharides were quantified using an ELS detector (Waters), and the concentration of each was calculated using a series of external standards (sucrose and melezitose). We could detect mono-, di-, and oligosaccharides but did not include polysaccharides in the current analysis. We also measured sugars in *A. nerii* honeydews from *A. incarnata* and *A. curassavica* in a pilot experiment conducted in December 2012; these data are presented for comparison in Appendix 2.

To measure honeydew amino acids, samples were resuspended in 50 μL 20 mM HCl, mixed with 50 μL of 0.5 mol/L borate buffer (pH 8.8), and derivatized with 26.4 μL of a 10 mmol/L aminoquinolyl-N-hydroxysuccinimidyl carbamate (AQC) solution (Cohen and Michaud 1993) in acetonitrile. Samples were filtered, and the derivatives were separated and quantified by high-performance liquid chromatography (HPLC) on a Waters HPLC (2690 Separations Module), using a Supelco Discovery HS C18 column (5.0 μm, 250 mm × 4.6 mm) with a C18 guard column. The gradient began at 100% A (pH 5.05 acetate buffer)/0% B (60% aqueous acetonitrile), and increased to 2% B at 0.5 min, 7% B at 10 min, and 45% B at 48 min, before returning to 0% B at 53 min. The flow rate was 1.0 mL/min, and the column temperature was 35°C. Peaks were detected with a Waters 996 photodiode array detector at the wavelength of maximum absorbance (248 nm). Standard curves were prepared with each amino acid to quantify amino acid concentrations from peak areas.

Cardenolides, sugars, and amino acids were separately transformed to percent weights by dividing the concentration of each compound by the total honeydew weight. Sugars and amino acid abundances displayed strong mean–variance relationships, which violate the assumptions of distance-based multivariate methods (Warton et al. 2012). The data were thus analyzed using generalized linear models (GLMs) implemented in the R package *mvabund* (Wang et al. 2012). For sugars, the model was fit to percent weight abundances with a negative binomial distribution. For amino acids, residual versus fit plots suggested that the data did not fit either a Poisson or a negative binomial distribution. We therefore classified amino acids as binary presence/absence data and compared plant species using a GLM with a binomial distribution, which provided a good fit. Percent weights of individual amino acids were then compared between plant species by pairwise Wilcoxon tests. For both GLMs, *P* values were calculated by pit trap resampling 999 times to account for correlation structure across abundances of individual peaks within samples, and univariate fits were adjusted for multiple tests (Wang et al. 2012). Ordination for visualization purposes was conducted using constrained correspondence analysis implemented in the R package *vegan* (Oksanen et al. 2013).

### Forced-diet experiment

To investigate ant colony responses to the honeydews produced by *A. nerii* aphids feeding on *A. curassavica* and *A. incarnata*, we forced colonies to feed on honeydew produced from one of the two plant species for 6 weeks. Each experimental ant colony began with one queen and 100 workers; we did not seed experimental colonies with any brood (eggs, larvae, or pupae). Each colony was assigned to a honeydew diet from either *A. curassavica* or *A. incarnata*. Replicate experimental colonies from the same source colony (*i.e.,* collected in exactly the same location) were assigned to each of the two plant species in a matched design. Three of the collection locations sourced two pairs each, and another two of the collection locations sourced three pairs each (*N* = 12 pairs). Colonies had access to one aphid-colonized plant and its associated honeydew at a time throughout the experiment. 9-week-old plants that had been seeded 5 days prior with five apterous adult aphids were introduced at 2-week intervals to experimental ant colonies (three plants per replicate over the 6-week experiment). In seven cases, aphid populations on *A. incarnata* plants started to crash before the end of the designated 2-week period. When this happened, the plant was replaced with another plant of the same age that had been seeded with aphids at least 5 days prior.

We did not manipulate aphid number, and aphid-colonized plants were harvested exactly 2 weeks after they were introduced to experimental ant colonies. To account for differences over time in aphid number between plant species, aphids were counted every other day throughout the 6 weeks. Plants were removed from the experimental bins after 2 weeks, and shoots with aphids were clipped at the base, placed in bags, and frozen at −20°C. Aphids were then removed from the shoots and separated. We also separated plant roots from soil and washed them in water. Shoots, roots, and aphids were dried at 45°C for ≥36 h before weighing.

Three behavioral assays were conducted on the ants in the course of the forced-diet experiment. In the first assay, instantaneous counts of ants foraging on plants were made twice per week at ∼14:00 h throughout the experiment. We counted ants foraging on the plant itself as well as ants walking across the chiffon mesh covering the top of the plant's pot (ants often appeared to collect honeydew from the mesh). Ants on the sides of the pot were not counted. In the second assay, beginning 1 week into the experiment, and at least once per week thereafter, we also made instantaneous counts of the number of ants recruiting to the 3:1 p:c artificial diet ∼45 min after placing the diet in the experimental bins. All of the ants that were within the 3-cm-diameter dish containing the diet were counted; most of these ants were visibly consuming the diet. Finally, in the third assay, we monitored ant exploratory response to an experimental structure, a tripod composed of three toothpicks with a paper platform, placed in the experimental bins. We conducted this test at least once per week throughout the experiment. All of the ants exploring the experimental structure were counted 10, 20, 30, and 40 min after placing the structure in the bin. Differences in the mean number of exploring ants between experimental treatments (*i.e.,* plant species) were in the same direction at all time points. Ants in both treatments were exploring in the highest mean numbers after 20 min, so we used the 20-min counts to compare treatments over time in a repeated-measure ANOVA in which the experimental replicate was included as a random effect.

To evaluate the effects of the plant honeydews on the survival, maintenance, and growth of ant colonies, we counted and weighed the colonies at the end of the experiment. After 6 weeks, we collected all ant queens, live workers, and brood (larvae and pupae), froze them at −20°C for 12 h, counted workers and brood, and dried everything at 45°C for ≥36 h. We then weighed ant queens and individual workers from each colony on a microbalance. Ant brood were too few and too small to weigh successfully. Two of our response variables, the number of live workers and the number of brood, varied significantly among source colonies (Tukey HSD *P* < 0.05), so these data were compared between plant species using matched-pair tests between replicates matched from the same source colony. All of the other analyses, including those for the behavioral assays, were conducted using plant species, the treatment, as a fixed factor.

### Choice experiment

To assess whether ants preferred aphid honeydew derived from *A. curassavica* or *A. incarnata*, we conducted a 1-week choice experiment. Experimental ant colonies for this experiment were composed of one queen and ≥20 workers (*N* = 15 replicates; 12 colonies with 30 workers and three colonies with 20 workers). Each colony had access to one aphid-colonized plant of each species throughout the experiment. Plants of both species were 4 weeks old at the start of the experiment and had been seeded 2 days prior with five adult apterous *A. nerii* aphids. Aphids were counted every day throughout the week, and we culled aphids to balance the total number of aphids on plants within experimental replicates. An ant nesting tube was placed in one half of each experimental bin, and the two aphid-colonized plants were placed in the other. We alternated placement of *A. curassavica* and *A. incarnata* among the replicates and within growth rooms.

To quantify ant preference, we counted the total number of ants visiting each plant in 2 min of continuous observation per bin, three times a day, for 1 week. Counts were made every day at 09:00, 13:00, and 17:00 h. Ants exploring or foraging on the plant itself or on the mesh covering the top of the plant's pot were counted. Individual ants that left the plant and came back within the 2-min observation period were recounted unless the experimenter had kept track of that individual. Because there were significantly more ants visiting plants at 09:00 h than at 13:00 h (*F*_2,87_ = 7.19, *P* < 0.002, Tukey HSD *P* < 0.05), the average number of ants visiting the two plant species during the week were compared by matched *t*-tests for each time of day separately. We also examined whether ant preferences changed over the course of the week by repeated-measures ANOVA.

## Results

### Honeydew chemistry

Aphids feeding on *A. curassavica* produced honeydew with significantly more cardenolides than aphids feeding on *A. incarnata* (Table 1). Cardenolides remained undetectable in *A. incarnata* honeydew even in samples 5Χ more concentrated than those used here (Appendix 1). The cardenolides present in the highest abundances in *A. curassavica* honeydew were of approximately intermediate polarity compared both to the total set of cardenolides found in the honeydew (Appendix 1). In contrast to total cardenolides, neither total sugars nor total amino acids differed significantly between the honeydews from the two plant species (Table 1).

**Table 1:** Percent (mean ± SE) honeydew dry weight of cardenolides, sugars, and amino acids produced by aphids feeding on *Asclepias curassavica* and *Asclepias incarnata*

	Aphids feeding on:				
Percent	*Asclepias curassavica*	*Asclepias incarnata*	Test statistic	df	Two-tailed *P*
Cardenolides	0.21 ± 0.05	0 ± 0	*Z* = −2.62	11	**0.009**
Sugars	31.6 ± 6.5	35.0 ± 8.4	*t* = 0.32	14	0.7
Amino Acids	2.3 ± 0.3	2.7 ± 0.4	*t* = 0.77	17	0.5

Bold values are significant.

**Figure 1:**
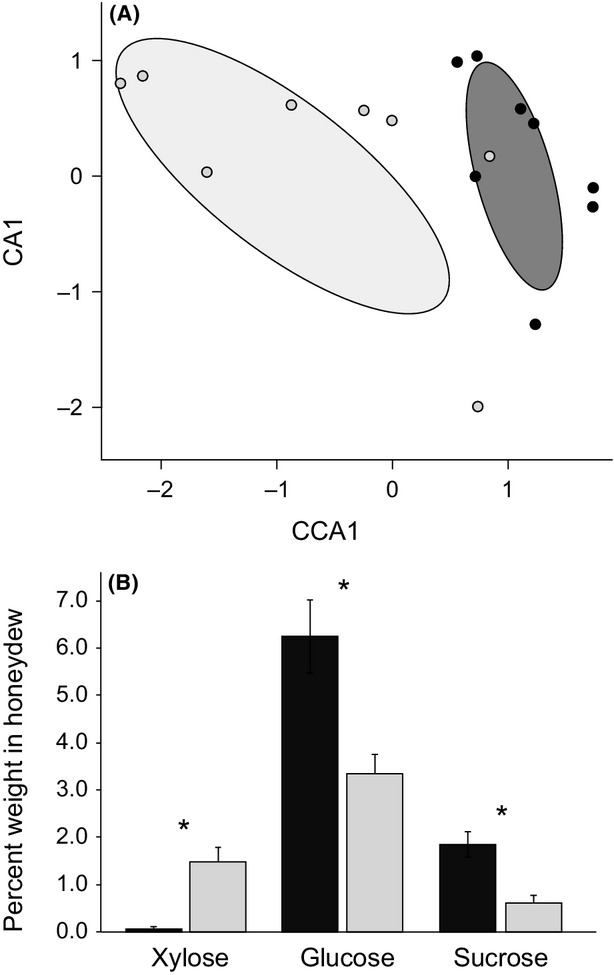
Multivariate analysis of honeydew sugar composition. (A) Relationship between the first unconstrained axis (CA1; eigenvalue = 0.50) and the axis constrained by host plant (CCA1; eigenvalue = 0.14) in a constrained correspondence analysis. Ellipses show 95% confidence intervals (function “ordiellipse” implemented by *vegan* in R). (B) Individual sugars that were significantly different between aphid honeydews produced from the two plant species by univariate generalized linear models. Bars indicate the percent dry weight of each sugar in the honeydew (mean + SE). Asterisk (*) indicates adjusted *P* < 0.04. In both panels, black represents *A. curassavica*; light gray represents *A. incarnata*.

Although the honeydews from the two plant species contained similar total sugars and amino acids, the composition of sugars and amino acids differed. The composition of honeydew sugars varied significantly between the two plant species (Fig. 1A; *D*_1,14_ = 44.05, *P* < 0.003). Overall, 17 sugar compounds were detected in aphid honeydews (Appendix 2). Three of these sugars contributed significantly to the difference between the two plant species, together explaining 66% of the total deviance: xylose (27%; *D*_1,14_ = 11.76, *P* < 0.008); sucrose (22%; *D*_1,14_ = 9.75, *P* < 0.02); and glucose (17%; *D*_1,14_ = 7.71, *P* < 0.04) (Fig. 1B). The composition of honeydew amino acids also varied significantly between the two plant species (*D*_1,10_ = 13.85, *P* < 0.03). We were able to quantify eight individual amino acids in the honeydews, including four essential amino acids (valine, isoleucine, leucine, and phenylalanine) (Table 2). In univariate binomial GLMs, only phenylalanine contributed significantly to the difference between the two plant species, explaining 55% of the total deviance (*D*_1,10_ = 7.64, *P* < 0.01). All of the essential amino acids were present in higher concentrations in honeydew from *A. incarnata* than from *A. curassavica*, and this difference was significant for isoleucine and phenylalanine (Table 2). However, the essential amino acids were present in much lower concentrations than the nonessential amino acids in honeydews from both plant species (essential vs. nonessential: *A. curassavica*: 0.09 ± 0.03 vs. 0.93 ± 0.17%; *A. incarnata*: 0.25 ± 0.05 vs. 0.64 ± 0.07%).

**Table 2:** Percent (mean ± SE) honeydew dry weight of individual amino acids produced by aphids feeding on *Asclepias curassavica* and *Asclepias incarnata*

	Aphids feeding on:				
Amino Acid	*Asclepias curassavica*	*Asclepias incarnata*	Test statistic	df	Two-tailed P*
Aspartic acid	0.07 ± 0.01	0.05 ± 0.02	*t* = −0.98	16	0.3
Glutamic acid	0.29 ± 0.11	0.12 ± 0.02	*Z* = −1.42	12	0.2
Serine	0.47 ± 0.11	0.26 ± 0.05	*t* = −1.50	17	0.2
Proline	0.19 ± 0.03	0.23 ± 0.02	*t* = 1.18	17	0.3
Valine	0.02 ± 0.01	0.03 ± 0.01	*Z* = 2.08	17	0.04
Isoleucine	0.00 ± 0.00	0.02 ± 0.01	*Z* = 2.89	16	**0.004**
Leucine	0.04 ± 0.02	0.11 ± 0.03	*Z* = 2.04	17	0.05
Phenylalanine	0.01 ± 0.00	0.09 ± 0.02	*Z* = 3.39	15	**0.0007**

* Bold indicates significance after Bonferroni correction for multiple comparisons (*P* = 0.05/8 = 0.006).

### Forced-diet experiment

The two plant species, *A. curassavica* and *A. incarnata*, exhibited differences in growth allocation and aphid population growth during the forced-diet experiment. *Asclepias curassavica* plants (*N* = 36) produced a higher shoot:root ratio than *A. incarnata* plants (*N* = 43) (aboveground dry mass =409.9 ± 15.5 vs. 50.7 ± 4.3 mg and root dry mass =119.6 ± 6.6 vs. 153.0 ± 13.4 mg, respectively). Consistent with this difference in aboveground biomass, aphid populations grew significantly larger on *A. curassavica* plants than on *A. incarnata* plants (mean ± SE = 150 ± 5 and 99 ± 5, respectively; *t* = −7.0, df = 22, *P* < 0.0001). Similar to the results from our preliminary experiments (see Methods), however, *A. incarnata* supported significantly more aphids per mg aboveground dry mass than *A. curassavica* (*A. curassavica*: 0.37 ± 0.02; *A. incarnata*: 2.05 ± 0.12; *Z* = 4.18, d = 22, *P* < 0.0001).

Importantly, there was no indication that the number of aphids or the quantity of honeydew determined ant responses. We did not quantify the differences in honeydew volume between plant species, but ants visited aphid-colonized *A. curassavica* and *A. incarnata* plants in similar numbers throughout the experiment (ants on plants in 43% vs. 42% of observations, respectively; *t* = −0.28, df = 22, *P* = 0.8). There was no relationship between the number of live ants at the end of the experiment and the average number of aphids on either plant species over the course of the experiment (*A. curassavica*: *F*_1,10_ = 2.62, *P* = 0.1; *A. incarnata*: *F*_1,10_ = 0.03, *P* = 0.9), suggesting that aphid honeydew was not a limiting resource for ant survival. Moreover, ants responded similarly to honeydew from each plant species in the pilot run of the experiment, in which *A. incarnata* and *A. curassavica* maintained statistically similar numbers of aphids (Appendix 3).

**Figure 2:**
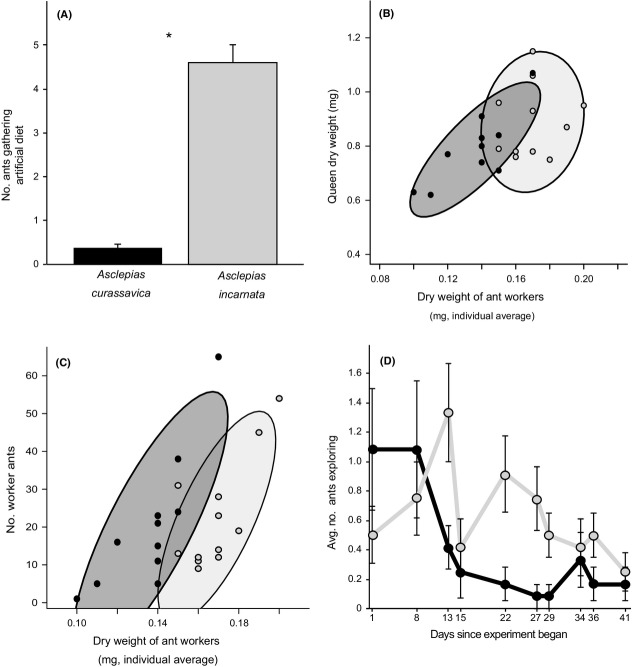
Results of the forced-diet experiment. (A) Number of ants (mean + SE) recruiting to and feeding from artificial diet for colonies feeding on *A. nerii* aphid honeydew derived from *A. curassavica* or *A. incarnata* plants. Asterisk (*) indicates a significant difference by two-tailed *t*-test (*t* = 10.39, d = 22, *P* < 0.0001). (B) Correlation between ant queen dry weight and the average weight of an individual worker in the same colony at the end of the experiment (*A. curassavica*: *r* = 0.79, *P* < 0.005; *A. incarnata*: *r* = 0.14, *P* = 0.7). (C) Correlation between the number of worker ants and the average weight of an individual worker in the same colony at the end of the experiment (*A. curassavica*: Spearman ρ = 0.77, *P* < 0.007; *A. incarnata*: Spearman ρ = 0.58, *P* < 0.05). For (B,C), ellipses represent 80% confidence distributions of the data points. (D) The average number of ants (mean ± SE) exploring an experimental structure introduced 20 min prior when feeding on aphid honeydew derived from *A. curassavica* or *A. incarnata* over time (repeated-measure mixed-effect ANOVA, plant species: *F*_1,22_ = 3.16, *P* = 0.051, time: *F*_1,214_ = 17.86, *P* < 0.0001, time × plant species: *F*_1,214_, *P* = 0.2). In all panels, black represents *A. curassavica*; light gray represents *A. incarnata*.

Honeydews produced from *A. curassavica* and *A. incarnata* differentially affected worker preference for artificial diet, queen and worker weight, the size of the colony, and worker behavior (Fig. 2). Ants feeding on *A. incarnata* honeydew were significantly more likely to gather the artificial diet than ants feeding on *A. curassavica* honeydew (Fig. 2A). This response was first recorded 7 days into the experiment and remained consistent throughout the next 5 weeks (repeated-measure mixed-effect ANOVA, plant species: *F*_1,22_ = 106.17, *P* < 0.0001; time: *F*_1,238_ = 0.05, *P* = 0.8).

At the end of 6 weeks, ant queens and workers feeding on honeydew from *A. incarnata* weighed significantly more than queens and workers feeding on honeydew from *A. curassavica* (Table 3). There was a significant, positive correlation between queen weight and worker weight for ants feeding on honeydew from *A. curassavica* but not for ants feeding on honeydew from *A. incarnata* (Fig. 2B). At the end of 6 weeks, there were marginally more live ant workers in colonies feeding on honeydew from *A. incarnata* and significantly more brood in colonies feeding on honeydew from *A. curassavica* (Table 4). All colonies decreased in size during the course of the experiment. There was a positive relationship between the number of live workers and average worker weight at the end of the experiment (Fig. 2C), which indicated that workers lost weight before dying and/or that smaller colonies raised smaller new workers. However, this relationship was stronger for ants feeding on *A. curassavica*-derived honeydew than for ants feeding on *A. incarnata*-derived honeydew (Fig. 2C), which is consistent with the above indications of greater nutritional stress on colonies feeding on *A. curassavica*-derived honeydew.

**Table 3:** Ant queen and worker dry weights (mean ± SE) at the conclusion of the 6-week forced-diet experiment

	Feeding on honeydew from:				
Weight (mg)	*Asclepias curassavica*	*Asclepias incarnata*	*t*	df	Two-tailed P
Queens	0.77 ± 0.04	0.89 ± 0.04	2.12	21	**0.05**
Workers	0.14 ± 0.01	0.17 ± 0.00	4.70	21	**0.0001**

Bold values are significant.

**Table 4:** Number (mean ± SE) of ant workers and brood (larvae and pupae) at the conclusion of the 6-week forced-diet experiment

	Feeding on honeydew from:					
Number	*Asclepias curassavica*	*Asclepias incarnata*	*S*	df	Two-tailed *P*	One-tailed *P*
Workers	19 ± 5	23 ± 4	−20.00	11	0.1	0.06
Brood	7 ± 3	3 ± 1	18.00	11	**0.008**	**0.004**

Data were compared by a matched-pair Wilcoxon Signed Rank test. High SEs reflect large differences among experimental colonies with different sites of origin, which were accounted for in the matched experimental design.

Bold values are significant.

Finally, throughout the latter 4 weeks of the experiment, workers feeding on *A. incarnata*-derived honeydew explored experimental structures in larger numbers than those feeding on *A. curassavica*-derived honeydew (Fig. 2D). The number of workers exploring the structure decreased over time for both plant species.

### Choice experiment

Ants displayed a marginal preference for *A. curassavica* honeydew over *A. incarnata* honeydew when they were given a choice (Table 5). Despite our efforts to maintain similar numbers of aphids on the two plant species by culling aphids once a day, and in contrast to aphid population growth in the forced-diet experiment, *A. incarnata* plants maintained more aphids than *A. curassavica* throughout the choice experiment (mean ± SE = 27 ± 1 and 18 ± 2, respectively; *t* = 5.10, df = 28, *P* < 0.0001). However, there was no relationship between the number of ants visiting a plant and the number of aphids on that plant (*A. curassavica*: *F*_1,13_ = 0.03, *P* = 0.9; *A. incarnata*: *F*_1,13_ = 0.13, *P* = 0.7).

**Table 5:** Number (mean ± SE) of ants on *A. curassavica* and *A. incarnata* plants in the choice experiment at different times of day

Time	*Asclepias curassavica*	*Asclepias incarnata*	df	*S*^1^	Two-tailed *P*	One-tailed *P*
Number of ants						
09:00 h	0.49 ± 0.11	0.49 ± 0.09	14	1.00	0.9	0.5
13:00 h	0.24 ± 0.07	0.15 ± 0.05	14	11.50	0.3	0.2
17:00 h	0.41 ± 0.09	0.24 ± 0.06	14	20.00	0.1	0.06
Number of ants/aphid						
09:00 h	0.03 ± 0.01	0.02 ± 0.00	14	11.50	0.3	0.1
13:00 h	0.01 ± 0.00	0.01 ± 0.00	14	5.50	0.6	0.3
17:00 h	0.03 ± 0.01	0.01 ± 0.00	14	32.00	**0.02**	**0.01**

1 Matched-pair Wilcoxon Signed Rank test.

Bold values are significant.

The highest number of ants visited both plant species at 09:00 h. At that hour, there was no difference in the total number of ants visiting the two plant species, but there were marginally more ants per aphid on *A. curassavica* than on *A. incarnata* (Table 5). More total ants visited *A. curassavica* than *A. incarnata* at both 13:00 h and 17:00 h, and this difference was marginally significant at 17:00 h (Table 5). In addition, at 17:00 h, there were significantly more ants per aphid on *A. curassavica* than on *A. incarnata* (Table 5). These ant responses were consistent over the week-long duration of the choice experiment for observations at 09:00 h and 13:00 h (repeated-measure mixed-effect ANOVA on total ants, time: *F*_1,178_ = 0.70, *P* = 0.4 and *F*_1,178_ = 1.42, *P* = 0.2, respectively). Preference for *A. curassavica* over *A. incarnata* increased marginally over the week for observations at 17:00 h (time: *F*_1,178_ = 3.65, *P* < 0.06).

## Discussion

The honeydew produced by clonal *A. nerii* aphids feeding on *A. curassavica* plants was chemically distinct from the honeydew the aphids produced on *A. incarnata* plants. Concentrations of cardenolides and of two of the most abundant sugars, glucose and sucrose, were higher in the honeydew derived from *A. curassavica*, whereas concentrations of xylose and of two of the four essential amino acids were higher in the honeydew derived from *A. incarnata*. These results are consistent with previous studies showing that hemipteran honeydew composition can be determined by the host plant genotype or species (Mittler 1958; Hendrix et al. 1992; Fischer et al. 2005). The presence of cardenolides in the *A. curassavica* honeydew but not in the *A. incarnata* honeydew suggests that the honeydews' chemical compositions reflected plant-species-specific differences: *A. curassavica* also contains higher concentrations of leaf cardenolides than *A. incarnata* (Sternberg et al. 2012).

Colonies of *L. humile* ants exhibited strong responses to this plant-derived variation in aphid honeydew chemistry (Fig. 2). Ants feeding on *A. incarnata-*derived honeydew were significantly more attracted to the artificial diet. The artificial diet contained more protein than carbohydrate (p:c = 3:1) because we expected the ants to gather the artificial diet in order to balance their intake of the carbohydrate-rich honeydew (Dussutour and Simpson 2008a). However, it is unlikely that *A. incarnata* colonies were more attracted to the artificial diet than *A. curassavica* colonies for the diet's protein content because, though scarce in the honeydews from both plant species, amino acids were actually present in higher concentrations in *A. incarnata*-derived honeydew than in *A. curassavica*-derived honeydew. Instead, ant preference for the artificial diet, whose carbohydrate content was 25% sucrose, inversely corresponded to the concentration of sucrose, and its glucose metabolite, in the aphid honeydews. Ants feeding on the low-sucrose *A. incarnata*-derived honeydew gathered more sucrose-containing artificial diet. Many species of ants have been shown to prefer sucrose to nearly all other sugars, and in particular to xylose (Völkl et al. 1999; Blüthgen and Fiedler 2004), which was relatively abundant in the *A. incarnata*-derived honeydew. If the *L. humile* ants prefer high-sucrose, low-xylose food sources, this would also explain the ants' moderate preference for *A. curassavica*-derived honeydew in the choice experiment.

Despite the ants' disinterest in the artificial diet when feeding on the preferred *A. curassavica*-derived honeydew, these colonies performed worse, in terms of queen weight, worker weight, and worker number, than ants feeding on *A. incarnata*-derived honeydew. There are at least two possible explanations for this result. First, the presence of cardenolides in *A. curassavica*-derived honeydew could negatively affect ant metabolism and thereby decrease ant weight (or larval growth) and survival. Cardenolides can have acutely toxic effects on the consuming animal or they can slow the animal's growth rate (Cohen 1983; Fukuyama et al. 1993; Agrawal et al. 2012). Although it has been suggested that cardenolides deter ants from feeding on honeydew in the field (Bristow 1991; Mooney et al. 2008; but see Molyneux et al. 1990), our results indicate that *L. humile* did not detect the differences in honeydew cardenolide content. Instead, the ants may have established their preferences based on the sugar content of the *A. curassavica*-derived honeydew, and then suffered the negative consequences of the concomitant increase in cardenolides.

A second, not mutually exclusive, explanation for the plant-species-specific difference in colony performance is that the ants' disinterest in the artificial diet when feeding on *A. curassavica*-derived honeydew deprived them of the nutritional resources they needed to succeed. If this were the case, it is unclear what may have triggered their disinterest. Ants recognize food sources based on chemical signals, and perhaps ants feeding on *A. curassavica*-derived honeydew did not recognize the artificial diet as containing necessary nutrients. Moreover, the hydrocarbon profiles of the ants themselves can change with different nutritional inputs (Liang and Silverman 2000), and with the ratio of protein to carbohydrate intake (Sorvari et al. 2008). It is unclear, however, why and how such possible changes in ant chemistry would affect the ants' diet consumption. Interestingly, although few ants feeding on *A. curassavica*-derived honeydew gathered artificial diet, colonies carried dead workers to the diet dish over the subsequent ∼12 h, which suggests that the ants recognized the diet (which was by then mostly desiccated) as an appropriate base for a midden pile. We also cannot rule out the possibility that the plants themselves caused changes in ant chemical cues; nesting material affects ant–ant recognition (Heinze et al. 1996), and prolonged foraging on a single plant spec-ies could create a similar effect. Future experiments could evaluate the effects of the individual compounds that varied between the plant species on ant nutritional preferences.

In contrast to the results for worker and queen performance, colonies feeding on *A. curassavica*-derived honeydew had significantly more brood than colonies feeding on *A. incarnata*-derived honeydew at the end of the forced-diet experiment. Although initially counterintuitive, this result is consistent both with possible sucrose deprivation of the ants feeding on *A. incarnata*-derived honeydew (Grover et al. 2007), if such deprivation was indicated by their greater interest in the artificial diet, and with negative feedbacks documented in other ant species between brood production and worker size and number (Porter and Tschinkel 1985). In addition, Nonacs (1991) reported that *Camponotus floridanus* colonies fed low-protein diets maintained higher pupal number and biomass than those fed high-protein diets and proposed that brood may serve as stable energy reserves for the colony when dietary protein is scarce. Similar rationale could explain our results because *A. curassavica* colonies consumed less protein than *A. incarnata* colonies by consuming less of the artificial diet.

In addition to performing better at the colony level when fed *A. incarnata*-derived honeydew, these workers were more exploratory in behavioral assays than workers feeding on *A. curassavica*-derived honeydew. Colonies feeding on *A. incarnata*-derived honeydew maintained more workers than colonies feeding on *A. curassavica*-derived honeydew, and colony size affects various aspects of ant behavior (Gordon 1987). In addition, workers maintained higher mass in *A. incarnata* colonies than in *A. curassavica* colonies, and worker size and colony size may interact to determine ant foraging behaviors (Howard and Tschinkel 1980). It was not possible to count workers until the end of the forced-diet experiment, so it is difficult to determine whether per capita exploratory activity increased concomitantly with overall colony activity, but such per capita effects can also result from changes in worker nutritional status (Grover et al. 2007; Pringle et al. 2011). Unexpectedly, different behaviors in the expl-oratory assays did not result in different numbers of ants visiting the two plant species, but the similar frequencies of plant visitation may have resulted from the oppos-ing effects of ant preference for *A. curassavica*-derived honeydew and greater numbers of workers in the *A. incarnata* treatment, and/or from the artificial limitation of providing the ants with access to only one plant at a time in the forced-diet experiment.

Overall, our results indicate that differences in honeydew composition can be derived from genetic differences between host plant species and that such differences can affect ant colony performance and behaviors. Because we did not examine phloem chemistry directly, we do not know how closely honeydew chemistry mirrors phloem chemistry. Aphids may produce different honeydews on the two plant species because they selectively metabolize or sequester phloem compounds that we did not observe in the honeydews (Mittler 1958; Douglas 1993), or because differences in phloem flow or viscosity between the two species creates osmotic differences in the aphid guts that result in different excreted compounds (Fisher et al. 1984). For example, the cardenolides present in *A. curassavica* honeydew exhibited nearly the full range of polarity present in *A. curassavica* leaves (Appendix 1) (de Roode et al. 2011; Sternberg et al. 2012), but the three most abundant cardenolides were of approximately intermediate polarity. Polar cardenolides may be more abundant in the phloem than less polar cardenolides because they are water soluble (Molyneux et al. 1990), and/or *A. nerii* may preferentially sequester less polar cardenolides for their own defense because they are more toxic (Botha et al. 1977). Whatever the specific chemical composition of the phloem, however, there were consistent, plant-species-specific differences in honeydew composition between distinct trials (Appendix 1 and 2) and despite considerable intraspecific variation.

Here we have shown that plants can exhibit interspecific differences in their indirect effects on ants via hemipterans. Differences in ant behavior that resulted from plant-derived differences in honeydew chemistry could feed back to affect plant fitness and community membership in the field. A next important step will be to examine whether plants also exhibit intraspecific variation in phloem traits subject to selection, which could lead to diffuse coevolution between plants and ants, mediated by aphid and non-aphid herbivores. Focusing on cardenolides as one important axis of variation in phloem and honeydew chemistry, cardenolide content in the common milkweed *Asclepias syriaca* has been shown to vary intraspecifically and exhibit ∼30% full-sib heritability (Vannette and Hunter 2011). In addition, *A. curassavica* itself exhibits geographic variation in cardenolide content (Rothschild et al. 1970), which suggests the potential for local adaptation in this trait. More generally, future studies of plant phloem chemistry should examine whether the presence of secondary compounds in phloem is the exception or the rule (Douglas 2006), and to what extent phloem chemistry is modified by hemipterans and their microbial symbionts (Katayama et al. 2013). Systems in which ants, plants, and hemipterans interact intensively and stably over time could be particularly informative for shedding light on questions of coevolution.

## Conclusions

We present evidence that plant-derived differences in the chemical composition of aphid honeydew can result from genetic differences between closely related plant species. Subtle differences between honeydews in the quantity of plant secondary compounds and in the composition of sugars, and perhaps of amino acids, were associated with differences in the performance and behaviors of honeydew-feeding ant colonies. In light of previous evidence indicating that hemipteran-tending ants have strong effects on plant fitness (Styrsky and Eubanks 2007), our results suggest the potential for diffuse evolution in plant–ant–herbivore interactions, mediated by plant traits underlying changes in phloem chemistry and plant responses to phloem-feeding hemipterans.

## Data Accessibility

Raw data: Dryad Digital Repository: http://doi.org/10.5061/dryad.7p2r4.

## Appendix 1. Cardenolides (mg/g) from two runs of honeydew collected in either April 2013 (Run 1^1^, pilot experiment) or October-November 2013 (Run 2^2^, this study)

**Table:**

		Rentention time (% of run time)						
Aphids feeding on:	Run	7	16	45	50	56	83	91
*Asclepias curassavica*	Run 1	0.09	0.18	0.54	0.98	0.75	0.10	0.03
	Run 2	0	0	0.68	0.43	1.03	0	0
*Asclepias incarnata*	Run 1	0	0	0	0	0	0	0
	Run 2	0	0	0	0	0	0	0

^1^Honeydew concentrations: *A. curassavica* (0.12–4.11 μg/μL); *A. incarnata* (0.20–3.49 μg/μL).

^2^Honeydew concentrations: *A. curassavica* (0.63–0.76 μg/μL); *A. incarnata* (0.61–0.70 μg/μL).

## Appendix 2. Percent (mean) honeydew dry weight of individual sugars from honeydew collected in either December 2012 (Run 1, pilot experiment, no ants) or October-November 2013 (Run 2, this study)

**Table:**

Aphids feeding on:	Run	Unidentified Peak 1	Unidentified Peak 2	Xylose	Unidentified Peak 3	Fructose	Glucose	Sucrose	Myo-inositol	Maltose	Unidentified Peak 4	Melezitose	Raffinose	Unidentified Peak 6	Unidentified Peak 7	Unidentified Peak 8	Unidentified Peak 9	Unidentified Peak 10
*Asclepias curassavica*	Run 1	2.87	7.06	2.66	0	3.33	5.23	2.89	0	2.11	3.29		1.19	1.50	1.10	1.43	0.17	2.19
	Run 2	5.98	9.39	0.06	0.03	5.36	6.25	1.85	0.07	0.53	0.01	0.10	0.22	0.01	0.39	0.61	0.57	0.14
*Asclepias incarnata*	Run 1	3.33	4.22	0.89	0	10.14	2.58	2.05	0.11	3.98	4.09		2.90	1.45	1.41	1.74	1.29	0.93
	Run 2	15.88	8.38	1.48	0	3.95	3.34	0.61	0.12	0.34	0.56	0.05	0.02	0	0.04	0.04	0.08	0.12

## Appendix 3. Results from pilot experiment.^1^ Number (mean ± SE) of aphids over the course of the experiment and of ant workers after 6 weeks of feeding on honeydew from one of the two plant species

**Table:**

	Plant species:					
Number	*Asclepias curassavica*	*Asclepias incarnata*	Test statistic	df	Two-tailed *P*	One-tailed *P*
No. aphids	111 ± 9	90 ± 10	*t* = −1.55	22	0.1	0.07
No. ant workers^2^	3.2 ± 1.2	4.4 ± 1.4	*Z* = −7.00	11	0.6	0.3
Queen weight (mg)	0.76 ± 0.05	0.83 ± 0.05	*t* = 0.97	15	0.3	0.2
Worker weight (mg)	0.14 ± 0.01	0.13 ± 0.01	*t* = −0.86	13	0.4	0.2

1 Colonies had access to an artificial diet different from that in this study, which ants in neither treatment appeared to gather, and many ants died in a mineral-oil barrier intended to keep them from nesting in plant soil.

2 Colonies began with one queen and 35 workers.
